# Supporting Older People to Live Safely at Home – Findings from Thirteen Case Studies on Integrated Care Across Europe

**DOI:** 10.5334/ijic.5423

**Published:** 2020-10-07

**Authors:** Manon Lette, Annerieke Stoop, Erica Gadsby, Eliva A. Ambugo, Nuri Cayuelas Mateu, Jillian Reynolds, Giel Nijpels, Caroline Baan, Simone R. de Bruin

**Affiliations:** 1 Amsterdam Public Health research institute, Department of General Practice, Amsterdam UMC, VU University Amsterdam, Amsterdam, NL; 2 National Institute for Public Health and the Environment, Bilthoven, NL; 3 Scientific Centre for Transformation in Care and Welfare (Tranzo), Tilburg University, Tilburg, NL; 4 Centre for Health Services Studies, University of Kent, Canterbury, UK; 5 Department of Health Management and Health Economics, Institute of Health and Society, University of Oslo, Oslo, NO; 6 Agency for Health Quality and Assessment of Catalonia (AQuAS), Barcelona, ES

**Keywords:** safety, risks, prevention, older people living at home, case study, integrated care

## Abstract

**Introduction:**

While many different factors can undermine older people’s ability to live safely at home, safety as an explicit aspect of integrated care for older people living at home is an underexplored topic in research. In the context of a European project on integrated care, this study aims to improve our understanding of how safety is addressed in integrated care practices across Europe.

**Methods:**

This multiple case study included thirteen integrated care sites from seven European countries. The Framework Method guided content analyses of the case study reports. Activities were clustered into activities aimed at identifying and managing risks, or activities addressing specific risks related to older people’s functioning, behaviour, social environment, physical environment and health and social care receipt.

**Results:**

Case studies included a broad range of activities addressing older people’s safety. Although care providers felt they sufficiently addressed safety issues, older people were often concerned and insecure about their safety. Attention to the practical and social aspects of safety was often insufficient.

**Conclusions and discussion:**

Integrated care services across Europe address older people’s safety in many ways. Further integration of health and social care solutions is necessary to enhance older people’s perceptions of safety.

## Background

Many people live at home well into old age. As people age, they are at increased risk to suffer from one or more chronic conditions, functional decline and frailty [1,2,3]. As a result, many older people face complex health and social care needs that require formal and informal support delivered by a wide range of primary and community health, social, long-term, acute and informal care providers [4,5]. In efforts to better address older people’s complex health and social care needs, care commissioners and service providers are increasingly adopting transformations towards integrated care [4,6]. Through these integrated care approaches, primary and community care providers aim to organise services so that they are person-centred, proactive, seamlessly joined-up across different care providers, and responsive to people’s multidimensional needs [6,7]. This is expected to contribute to higher quality care and support, that is safe, effective, timely and respectful of people’s individual preferences [4,5,8]. Integrated approaches to primary and community care are considered essential in supporting older people to live at home [9].

To support older people living at home, it is essential to maintain their safety. Following the Institute of Medicine’s publication ‘To Err is Human’ [10], research on patient safety has predominantly focused on the “prevention of errors and adverse effects associated with health care” [11, par. 1]. However, older people may experience a wide range of limitations in multiple domains of life that could also pose risks to their ability to live safely at home. Such risks could be related to their health and functional capacities, as well as to their social contexts and physical environments [12,13,14,15,16,17,18,19]. This observation implies the need for a broader perspective on the safety of older people living at home. Lau et al. (2007) [19] proposed a model for health-related safety that extends beyond the traditional definition of patient safety and incorporates this wider range of risk factors. Studies have shown, for example, that older people with functional limitations, cognitive problems or low psychosocial resources are at increased risk of unwanted outcomes such as avoidable hospital admissions [20,21,22]. Therefore, it is important to focus efforts on the mitigation of such risks and the prevention of problems.

The comprehensive, interdisciplinary and proactive nature of integrated care provides opportunities for addressing problems and risks to older people’s safety at home [23,24]. However, safety as an explicit aspect of integrated care for older people living at home warrants more research attention [24,25]. Recognising the need for integrated care approaches to address safety, a cross-European project on integrated care for older people living at home included safety as one of its key areas of interest [7]. The four-year EU funded project, called SUSTAIN (Sustainable Tailored Integrated Care for Older People in Europe), included thirteen sites across Europe that aimed to improve their integrated care services for older people. The experiences and insights gained from collaborating with these integrated care sites provide a unique opportunity to improve our understanding of how safety risks for older people living at home are addressed by integrated care services across different countries and contexts in Europe. Therefore, the first aim of this study is to identify the activities implemented by the integrated care sites to address and manage safety risks of older people. The second aim is to describe experiences with risk management in older people’s home situation from the perspectives of health and social care providers, older people and their informal caregivers. The knowledge gained from this study, based on experiences from across all the SUSTAIN sites, might help to develop recommendations for improving integrated care from a safety perspective that could be transferable across multiple contexts and health and social care systems.

## Methods

**Figure 1:**
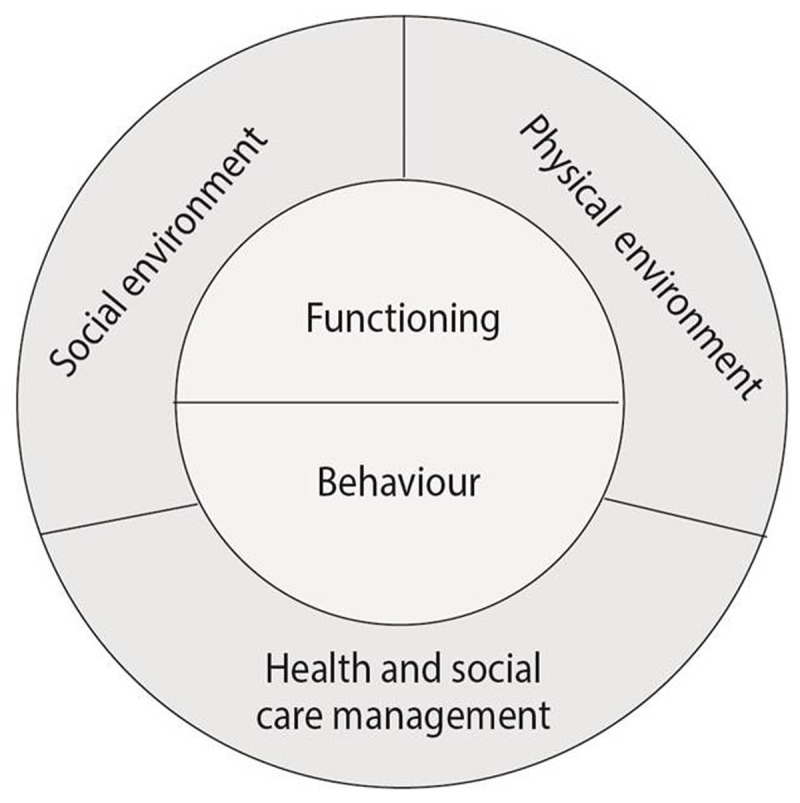
Framework for the conceptualisation of safety, showing the different domains of safety risks for older people living at home [23]. This framework was partly based on the principles of Lau at al.’s (2007) framework for health-related safety [19].

This paper describes the results of an overarching content analysis of thirteen case studies on integrated care sites throughout Europe. These case studies were developed during the SUSTAIN project, and for this particular study, they were analysed with a focus on safety. To understand safety in the context of integrated care for older people living at home, we used a framework that conceptualises safety as preventing or reducing the risk of problems that could undermine older people’s ability to live independently at home (see Figure 1) [23]. Following the principles of Lau et al.’s (2007) model for health-related safety, which was introduced in the background section of this paper [19], this framework incorporates risks related to individual functioning and behaviour (e.g. cognitive decline, poor diet, physical inactivity), social and physical environments (e.g. caregiver burden, economic hardship, hazards in the home), and health and social care management (e.g. polypharmacy, fragmentation of care) [12,13,14,15,16,17,18,19]. The following sections will provide more background on the SUSTAIN project, elaborate briefly on the development of the case studies and describe the procedures used for this overarching case study analysis focused on safety.

### The SUSTAIN project

The SUSTAIN project adopted a multiple embedded case study design [26] to evaluate and compare improvement processes of different integrated care sites across Europe. More details on the design of the SUSTAIN project can be found elsewhere [7]. In summary, thirteen sites participated, which were located in Austria, Estonia, Germany, Norway, Spain (Catalonia), the Netherlands and the United Kingdom. All integrated care sites focused on older people living at home with complex care needs; however, they provided different types of care services within different national and local settings. Following an action research approach, stakeholders from the participating sites (e.g. health and social care providers, municipalities, representatives of older people) closely collaborated with SUSTAIN research partners to design and implement improvement projects tailored to local priorities [27]. They carried out a wide range of activities focused on the project’s key areas of interest, namely: safety, prevention-orientation, person-centeredness and efficiency. Table 1 provides an overview of the participating sites and the objectives of their improvement activities.

**Table 1:** Characteristics of thirteen integrated care sites participating in the SUSTAIN project (adapted from de Bruin et al., 2018 [7] and de Bruin et al., 2018 [28]).

Country	Region	Integrated care site	Type of care services	Improvement project objective

**Austria**	Vienna	Gerontopsychiatric Centre	Dementia care	To improve detection of dementia and case- and discharge management of hospitalised people identified with a cognitive disorder.
**Estonia**	Ida-Viru	Alutaguse Care Centre	Home nursing and rehabilitative care	To develop a person-centred way of working by engaging older people, informal caregivers and a multidisciplinary care team in the process of defining a goal-directed care plan.
	Tallinn	Medendi	Home nursing	To increase the engagement of the older person, informal caregiver and different professionals in the development of a joint care plan, and to support information exchange between the older person, informal caregivers and professionals about the older person’s situation, needs and objectives.
**Germany**	Uckermark	KV RegioMed Zentrum Templin	Rehabilitative care	To enable people with care needs (including people who completed a complex therapy program) to receive the right services, by providing information and advice on available care and support services.
	Berlin Marzahn-Hellersdorf	Careworks Berlin	Home nursing and rehabilitative care	To improve inter-professional case management and multidisciplinary collaboration between general practitioners, (para)medical therapists and nurses by transferring prescription-competence from General Practitioners to (para)medical therapists and nurses; and to establish formalised interactions and communication space among involved (formal and informal) caregivers.
**Norway**	Surnadal	Surnadal Holistic Patient Care at Home	Home nursing and rehabilitative care	To expand and improve healthcare services delivered at home.
	Søndre Nordstrand in Oslo	Søndre Nordstrand Everyday Mastery Team	Rehabilitative care	To increase people’s sense of personal control, reduce reliance on traditional care services and maintain and encourage good functional ability and social participation among older people.
**Spain (Catalonia)**	Osona	Severe Chronic Patients/Advanced chronic disease/Geriatrics Osona	Proactive primary and intermediate care	To improve person-centeredness of care by conducting a standard, multidimensional joint assessment and elaborating a shared individualised care plan among involved health care and social care professionals and the older people and informal caregivers.
	Sabadell	Social and health care integration Sabadell	Proactive primary care	To establish a systematic, multidimensional assessment and care plan tailored to multiple health and social care needs of each older person and to establish care plans that people feel knowledgeable and active about, targeted at those unknown to social services.
**The Netherlands**	West-Friesland	Health and social care West-Friesland	Proactive primary care	To improve collaboration between General Practitioners and practice nurses, case managers for people with dementia and the social community team in order for them to adequately address older people’s health and social care needs; and to improve professionals’ person-centred way of working.
	Arnhem	Good in one Go	Transitional care	To clarify and align the various scenarios of a sudden need for more intensive care of a person living at home in a crisis (such as dementia or brain injury).
**United Kingdom**	Kent	Over 75 Service	Proactive primary care	To keep older people with long-term conditions and complex care needs at home independently for as long as possible and to improve care coordination across existing services around these people.
	Kent	Swale Home First	Transitional care	To ensure medically optimised hospitalised people are able to be discharged straight home with the right support and to make the person’s discharge smoother, quicker and safer by moving to a single assessment.

### Case studies

Throughout the project, SUSTAIN researchers in each country collected and analysed information on the integrated care sites’ way of working, as well as the development and implementation of improvements to this way of working. Each participating integrated care site was considered one case study. The development of the case studies was characterised by a structured approach, using standardised tools and procedures for data collection, analysis and reporting. This was done to ensure methodological consistency across the different case studies. Case studies were analysed using rigorous methods, which were based on triangulations of multiple qualitative and quantitative data sources and guided by predefined propositions [26]. Furthermore, case studies paid specific attention to the local and country context, ensuring a close connection between data and context. More detailed information on the methodological development of the case studies is provided in Appendix 1.

The results of each case study were reported in different documents. These documents included the following:

1. A baseline report [29] which included descriptions of the characteristics, problem analysis, stakeholder analysis, and improvement plans for each case study. This report is publicly available on www.sustain-eu.org.
2. The improvement and implementation plans that were developed in each integrated care site.
3. The case study reports [30,31,32,33,34,35,36] describing the improvement processes of the participating integrated care sites. These reports included information regarding experiences with integrated care activities from different perspectives (i.e. those of health and social care providers, older people and informal caregivers). These reports are publicly available on www.sustain-eu.org. In order to ensure a comprehensive and accurate understanding and assessment of the case studies, data from the case study reports was cross-checked with data from the sites’ individual data sources (see Appendix 1). Pertinent additional data gathered from this cross-checking process was included in the current analyses as well.

### Content analysis

The overarching content analysis of the documents reporting the thirteen case studies was guided by the Framework Method for analysis of qualitative data [37]. A coding framework was developed using a deductive approach, in which the conceptualisation of safety, as shown in Figure 1, as well as this study’s two research aims determined the main and sub-codes. This coding framework was tested on part of the data, after which an additional main code was added inductively. The finalised coding framework, which is shown in Table 2, was used to identify and categorise safety-related information in the different documents reporting case study results. In line with the coding framework and the aims of this paper, this safety-related information consisted of either activities to address and manage risks, or respondents (e.g., health and social care providers, older people) experiences with these activities. Because the aim of the paper was to identify all activities addressing older people’s safety, the coding process did not distinguish pre-existing activities from activities implemented during the improvement processes.

**Table 2:** Analysis framework used for content analysis of data sources.

Main codes	Sub-codes

Identifying and managing risks*	Activities Experiences from older people and their informal caregivers Experiences from health and social care professionals and managers
Addressing risks deriving from older people’s functioning	Activities Experiences from older people and their informal caregivers Experiences from health and social care professionals and managers
Addressing risks deriving from older people’s behaviour	Activities Experiences from older people and their informal caregivers Experiences from health and social care professionals and managers
Addressing risks deriving from older people’s social environment	Activities Experiences from older people and their informal caregivers Experiences from health and social care professionals and managers
Addressing risks deriving from older people’s physical environment	Activities Experiences from older people and their informal caregivers Experiences from health and social care professionals and managers
Addressing risks deriving from older people’s health and social care management	Activities Experiences from older people and their informal caregivers Experiences from health and social care professionals and managers
General experiences with safety for older people living at home	Experiences from older people and their informal caregivers Experiences from health and social care professionals and managers

* This theme was identified inductively after reviewing the data.

Two researchers (ML and AS) extracted information from the different documents reporting on the case studies, and they independently coded this information using MAXQDA (version 2018-0-5). The researchers cross-checked each other’s coding, and discussed potential differences in order to reach consensus. Coded data was then examined in order to identify and define recurring patterns and themes. Draft findings were discussed among all authors throughout the analysis process.

### Ethical considerations

Ethical review committees of Estonia, Norway, Spain (Catalonia) and the United Kingdom provided ethical approval of the SUSTAIN project. In Austria, Germany and the Netherlands, national standards and regulations allowed for the exemption of research activities from the need for ethics committee review. Informed consent was obtained for all study participants prior to data collection.

## Results

The participating integrated care sites addressed safety in a number of ways. Although only one site (Swale Home First) explicitly mentioned safety in its objective (to ‘make the person’s discharge from hospital smoother, quicker and safer’) (see Table 1), all sites addressed safety concerns within their ways of working by using a proactive and prevention-oriented approach. These approaches aimed to, for instance, enhance older people’s self-management abilities, prevent deterioration of chronic conditions, avoid crises and mitigate specific risks such as medication errors or falls. The following two sections will provide an overview of the activities addressing older people’s safety identified in the case studies, and an overview of experiences with safety-related activities from the perspectives of older people, informal caregivers and health and social care providers.

### Safety-related activities identified across integrated care case studies

**Figure 2:**
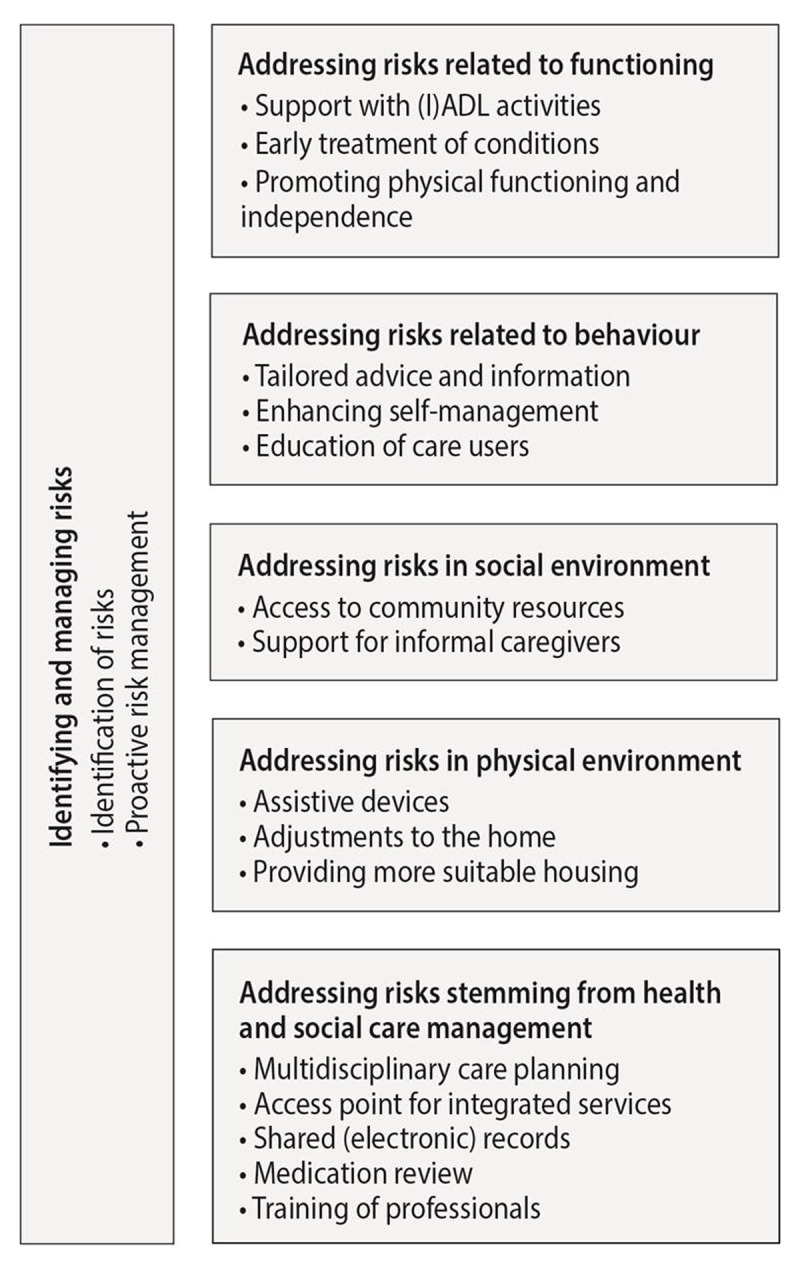
Activities addressing safety risks as identified in the participating integrated care sites.

Figure 2 provides an overview of all activities addressing safety of older people living at home that were identified in the case studies. Activities were clustered into six different categories, according to the aspect of safety on which the activity focused. The first category comprised generic activities aimed at identifying and managing risks. The other five categories included activities addressing specific risks related to older people’s 1) functioning, 2) behaviour, 3) social environment, 4) physical environment and 5) health and social care receipt.

#### Activities identifying and managing risks

In all but one site, generic activities that focused on the identification and management of risks were identified. Early identification of risks was enhanced by performing multidimensional assessments, which allowed health and social care providers to identify risks on various domains of life. Activities that aimed to promote proactive risk management included, for example, regular home visits during which health and social care providers could monitor changing risks and needs over time in people’s own home environments. Furthermore, risks were managed through the development of a comprehensive care plan wherein both health and social care providers and older people could plan for existing and future care needs.

#### Activities addressing risks related to older people’s functioning

Ten out of thirteen sites addressed risks related to people’s functioning. Such risks included their physical and cognitive limitations, deteriorating health conditions and fall risk. Activities addressing these risks aimed to either improve older people’s functioning or prevent functional decline. Sites often provided support with (Instrumental) Activities of Daily Living ((I)ADL), such as home maintenance, bathing and dressing. For many older people, this support was necessary in order for them to be able to live independently at home. Other activities addressing functional limitations included physical and occupational therapy, exercise classes, pain management and early treatment of health conditions. Furthermore, some sites promoted older people’s personal control and independence by providing rehabilitative services.

#### Activities addressing risks related to older people’s behaviour

Eleven sites targeted older people’s behaviour by addressing risks related to, for example, lifestyle, self-care or dependency. Many sites included activities that consisted of providing information and advice. For some sites, this was only described in general terms (i.e., providing safety advice), but other sites provided tailored advice and counselling on how to manage and cope with specific risks. Advice addressed, for example, healthier habits (e.g. diet, physical activity) or coping with potential hazards in people’s homes. In one site, workshops were organised in order to educate people on the process of growing older, including accepting and managing one’s own limitations in order to avoid potential risks.

#### Activities addressing risks related to older people’s social environment

All but one site addressed risks related to people’s social environment, which included risks such as social isolation, loneliness and caregiver burden. In seven sites, health and social care providers gave older people advice on social support or directed them to community resources, such as low-threshold activities with a social component (e.g. day care services, befriending services). Such activities aimed to improve people’s emotional and social functioning. Some sites addressed caregiver burden by providing support for people’s informal caregivers. Case studies showed that many of the recruited older people were dependent on their informal caregivers, and caregivers were often spouses who were also experiencing functional limitations. Support activities for informal caregivers included assessments of informal caregivers’ own needs, advice on how to care for an older person, and providing respite options by arranging day care or temporary stay services.

#### Activities addressing risks related to older people’s physical environment

Ten sites addressed older people’s physical environment. Most of these sites provided assistive devices, such as mobility aids and personal alarms, or enabled adjustments to people’s homes, for example by placing handgrips, rails or stair lifts. These activities were either provided by the integrated care site itself, or health and social care providers from the site advised older people on these matters and referred them to organisations who delivered such services. In one site, health and social care providers also discussed options of moving to more suitable housing with older people.

#### Activities addressing risks related to health and social care delivery

All sites addressed risks related to health and social care delivery. First, sites addressed fragmentation of services, thereby enabling a multidimensional approach to safety and preventing continuity gaps and errors caused by communication failures. For instance, all sites incorporated activities that enhanced a multidisciplinary approach in care planning. Examples of such activities included promoting professionals’ knowledge of available services, organising multidisciplinary consultations or using a common care planning tool across disciplines. Half of the sites employed activities to optimise an access point for integrated services by appointing a case manager, or naming specific professionals as key contact persons. Furthermore, information sharing across professionals was established through shared (electronic) records or by enhancing formal and informal communication processes between professionals. Second, most sites addressed risks related to the use of (multiple) medications by performing medication reviews, addressing treatment adherence, and providing medication dispensers. Finally, some sites addressed risks related to lack of knowledge and skills among health and social care providers. Specifically, by organising training for providers that targeted, for example, their knowledge of specific conditions or their provider-patient communication skills, these sites aimed to improve their ability to provide adequate care and support.

### Experiences with safety across SUSTAIN sites from the perspectives of health and social care providers, older people and informal caregivers

#### A proactive and multidimensional approach was valued for understanding risks

Case studies suggested that health and social care providers perceived safety to be a natural aspect of their professional disciplines, but they did not always consider safety an element explicitly important to integrated care. Findings from the case studies revealed that a multidisciplinary and proactive approach enhanced the identification of risks before an adverse event occurred, and enabled follow-up and coordination of care activities. Multidimensional assessments in people’s home environments helped professionals to gain a better understanding of risks and enabled them to provide more tailored support. This multidisciplinary approach was also valued by older people, as was the availability of a case manager or a key contact person. These activities enhanced older people’s perception of care continuity, which made them feel safe and well cared for.

#### Experiences with communication between care providers and older people were mixed

Case studies suggested that communication and information exchange between older people and health and social care providers was not always adequate. For example, while health care providers considered medication reviews an important aspect of safe care, older people were not always aware of or involved in such reviews. Furthermore, health and social care providers generally felt that they addressed the safety of older people living at home in an adequate manner. However, they did experience that older people were sometimes reluctant to plan ahead and anticipate worsening conditions. Older people, on the other hand, perceived that health and social care providers were not always proactive in providing safety-related information and support. Overall, case studies indicate that older people often felt concerned and insecure about their ability to live safely at home.

#### Addressing social risks was experienced as complex, but relevant for older people

Although most sites used a multidimensional approach to care, case studies suggested that at times, care and support focused primarily on physical functioning and less on emotional and social risks. In some cases, this lack of attention to social risks was attributed to the advanced health conditions of some older people. However, case studies also highlighted that professionals did not always have up-to-date information about available community resources, which made it difficult for them to provide or refer older people to these types of services and support. Still, when social support was provided within integrated care sites, older people gained confidence and felt empowered by such support.

#### Support for informal caregivers was insufficient

An additional aspect of safety that was highlighted in the case studies was the lack of information and support for informal caregivers. Caregivers were often challenged by insufficient knowledge of services available in the community, or lack of support with overseeing older people’s medication use – e.g. information about potential problems or side effects. Overall, case studies indicated that support for informal caregivers could be more of a priority within integrated care sites, as caregivers were found to play an important role in supporting older people to live safely at home.

## Discussion

This study provided insight into how safety of older people living at home was addressed in thirteen integrated care sites across Europe. Although safety was generally not an explicit objective of the sites, the findings from this study showed that a broad range of the activities undertaken were aimed at mitigating specific risks, preventing (avoidable) deterioration of functioning, and enhancing older people’s ability to manage their lives at home. That said, experiences across the case studies also indicated that while health and social care providers thought that their activities had sufficiently addressed safety issues, older people and their informal carers often still felt concerned and insecure about how to live safely in their homes. This suggests that providers and older people may have different perceptions of what is necessary for older people to live safely at home. Specifically, our study showed that providers tend to focus primarily on safety aspects related to older people’s clinical and physical functioning, as well as on risks associated with the care they provide. The focus on such risks was shown, for example, in activities pertaining to managing health conditions, reducing fall risk, reviewing medications and ensuring care continuity. However, in line with previous research, older people considered practical support with (I)ADL, social support, and a safe and accessible home and living environment to be important factors for managing safely at home [38,39,40,41]. Targeting these practical, social, and environmental aspects of safety might be crucial for improving older people’s perceptions and experiences of safety.

In addition to identifying activities targeting specific risks, this study also distinguished several more general risk management approaches that relate to integrated care elements such as a comprehensive approach, care coordination, proactiveness and person-centredness. For example, comprehensive assessments by an interdisciplinary health care team were valued by health and social care providers as well as older people. A comprehensive understanding of risks is essential, given the multidimensional nature of safety for older people living at home. It is important to note that, while this study categorised safety-related activities into separate domains, some activities may address risks in multiple domains simultaneously. For example, participation in exercise classes may improve people’s physical functioning whilst also addressing social isolation [42]. Furthermore, the risks themselves may also be addressed through multiple types of activities. For instance, the risk of falling can be targeted by addressing people’s physical functioning as well as their physical environment [43]. This interrelatedness of different domains of safety implies that the manner of addressing safety risks should be multifactorial. To support such a comprehensive and multifactorial approach, care coordination across professionals from different disciplines is therefore essential in order to adequately support safety of older people living at home.

Additionally, this study showed that many of the safety-related activities identified across the case studies entailed proactive activities that enabled providers to identify and tackle risks in a timely manner. This is important since proactive and prevention-oriented approaches are imperative in order to maintain older people’s safety [16,18,38]. However, as also suggested by previous studies, our findings showed that older people may not always be receptive to proactive detection of risks and needs – that is, they are not always willing to anticipate future needs and can perceive attempt to encourage this as patronising [38,44]. Confidential relationships between older people and professionals, which are built on trust and tailored to people’s needs, seem to be key to the success of such a proactive approach to care and support [38,44,45,46]. Enabling professionals to cultivate such relationships, and ensuring they have adequate time to provide advice and communicate with older people about preferences and experiences, should therefore be an important aspect of integrated care services that aim to effectively address older people’s safety. The above observations link older people’s safety to critical elements of integrated care, thereby underpinning the value of integrated care in addressing the safety of older people living at home.

### Methodological considerations

The SUSTAIN project, which is the source of data for this study, provided the opportunity to use in-depth data on real-world examples of integrated care sites across different contexts in Europe. The project’s multiple case study design allowed us to gain insights into experiences with safety and safety-related activities from multiple perspectives. However, some limitations should be considered. First, it should be noted that our data did not allow us to draw conclusions on the effectiveness of safety-related activities, nor were we able to clearly distinguish experiences with pre-existing activities from activities implemented during improvement projects. Second, the focus on safety adopted in the analyses for this study did not necessarily reflect the emphasis that was placed on safety by the participating integrated care sites. Although it was one of the SUSTAIN project’s key areas of interest, participating integrated care sites addressed safety in different ways according to their local priorities. Third, integrated care sites may have had varying understandings of safety. Despite project-wide efforts to increase consistency across sites, including shared operationalisations of the key areas of interest, we cannot exclude the possibility of some remaining variation in the case studies’ interpretation of safety. For this specific study, therefore, we developed one conceptual framework of safety, which we applied similarly to all case studies. This way, we were able to identify and analyse safety-related information in a comparable way.

The development of the case studies also presented some methodological challenges. First, research partners were involved in data collection and analyses, and they also closely collaborated with local stakeholders to facilitate improvement processes. This dual role had the potential of introducing risks to the case studies’ methodological rigour [7]. The SUSTAIN project addressed this by distinguishing between two types of research partners: local research partners who were involved in the individual case studies, and research partners who coordinated the overarching analyses. Throughout the project, these coordinating research partners regularly consulted the local research partners about their case studies to reinforce their scientific distance from the data and stimulate critical reflection on their role in the research process. This way, the SUSTAIN project enhanced transparency and accuracy of findings, while at the same time recognizing and accepting that findings were context-bound.

Furthermore, the involvement of many different local research partners, who had varying backgrounds and worked in different local and national contexts, posed challenges to the methodological consistency across case studies. The SUSTAIN project strived to mitigate these challenges in various ways. Local research partners involved in the case studies were expected to use standardised tools and templates for data collection, analyses and reporting. Furthermore, the coordinating research partners reviewed the documents provided by the local research partners for internal consistency of data collection and analyses. Aside from some minor variations in the manner in which data was collected, no significant differences in methodological approach were found, which indicates a satisfactory degree of consistency across case studies.

### Implications for research, policy and practice

The findings of this study provide some indications for activities that worked well and things that could be improved in order to support older people to live safely at home. In doing so, it highlighted some areas that might benefit from specific components of integrated care. In line with findings from previous studies [18,47,48], this study also suggests that lack of care continuity and provision of insufficient or conflicting information negatively influenced older people’s safety. Components of integrated care approaches that address communication and collaboration across different care interfaces, such as case management or multidisciplinary care planning, may, therefore, help to safeguard older people’s ability to live safely at home [49,50,51]. Furthermore, this study suggests that home visits helped professionals to assess risks in the context of people’s daily lives. Studies have shown that incorporating home visits into integrated care programs may also be a good way to build trusting relationships between professionals and older people that are necessary for a proactive care approach [52,53]. A multidimensional approach to addressing risks was also found to be important for older people as well as care providers. In many cases, the successful implementation of such an approach requires that several barriers are addressed, such as challenges in referring older people to available community resources [54,55] and financial constraints in procuring assistive devices [18,38]. Moreover, attention to the concerns and needs of informal caregivers was considered insufficient in many of the integrated care sites. By providing support for informal caregivers, care providers could ensure a strong social support network that can improve older people’s ability to live safely at home [18].

Overall, many similarities were observed in safety-related activities and experiences across the different integrated care sites. Even though the characteristics of integrated care sites and the contexts within which they operated were very diverse, similar efforts were being undertaken. These commonalities might inspire other policy makers and professionals across different settings and health and social care systems to improve their integrated care practices from a safety perspective. At the same time, it should be recognised that many of these good practices are complex interventions that are set in complex systems [56,57]. The activities identified across the different integrated care sites are not isolated practices but reside in an existing integrated care system. Transferring these practices to other integrated care systems requires time, effort and financial support in order to tailor them to the local context. Still, exchange of evidence and experiences on practices that worked and did not work in different contexts can foster further development of integrated care approaches. The overview provided in this study provides starting points to what might be critical functions in integrated care for promoting the safety of older people living at home. Besides its potential for inspiring improvement efforts, this overview might also serve as a guide for further developing sets of process quality indicators for the evaluation of integrated care approaches [58].

## Conclusion

This multiple case study aimed to provide insight into how integrated care sites across Europe address the safety of older people living at home. By reporting on an overarching level, this study provides insight into the full range of potential activities across different contexts and countries. Our findings indicate that addressing safety for this population is a common practice among integrated care sites, and similarities in observed activities and experiences imply the applicability of these findings across different settings and contexts. Although the proactive and interdisciplinary nature of integrated care services has the potential to address older people’s safety in a comprehensive way, more attention to the practical and social aspects of safety is needed to better fulfil the needs of older people. Continued efforts to integrate medical and non-medical solutions to safety are therefore necessary.

## Additional File

The additional file for this article can be found as follows:

10.5334/ijic.5423.s1 Appendix 1. Methods used in individual case studies – tools and procedures for data collection, analysis and reporting.
