# Supplementary material for: Supporting Older People to Live Safely at Home – Findings from Thirteen Case Studies on Integrated Care Across Europe
Source: Int J Integr Care. 2020 Oct 7;20(4):1. doi: 10.5334/ijic.5423 (PMC7546110; doi:10.5334/ijic.5423)
Supplement: Appendix 1. — Methods used in individual case studies – tools and procedures for data collection, analysis and reporting. [file ijic-20-4-5423-s1.pdf]

Appendix 1: Methods used in individual case studies - tools and procedures for data collection, analysis and reporting

Manon Lette, Annerieke Stoop, Erica Gadsby, Eliva A. Ambugo, Nuri Cayuelas Mateu, Jillian Reynolds, Giel Nijpels, Caroline Baan, Simone de Bruin

SUSTAIN aimed to support and monitor improvement processes of established integrated care sites in order to attain improvements in the project's key areas of interest, being person-centeredness, prevention-orientation, safety and efficiency. As described elsewhere [1], the project consisted of three distinct phases, during which different types of data were collected and analysed. A description of these phases and the resulting data is provided below.

Preparation phase: During the preparation phase, which started in autumn 2015, research partners established working relationships with the participating integrated care sites and carried out baseline assessments. For these baseline assessments, researchers from each participating country gathered information on each site's key characteristics, way of working and areas of current practice that might be subject to improvement. Three types of data sources were used to this end:

1. Interviews: For each integrated care site, semi-structured interviews were conducted with one older person receiving services from the site, one informal caregiver, one health or social care professional involved in the site and one manager of the site. Interviews covered the context and characteristics of the site, stakeholders' interpretation of SUSTAIN's four key domains, facilitators and barriers to integration, and potential areas for improvement. Semi-structured interview schedules were developed at Consortium level and used by all research partners in order to ensure uniformity.
2. 'Characteristics of integrated care site'-tool: For each site, managers were additionally asked to provide quantitative information on key aspects of their integrated care sites, such as their rationale, aims and objectives, structure and outcomes, and to identify key stakeholders relevant for the sites.
3. Workshop meetings: For each site, identified key stakeholders participated in workshop meetings to discuss the outcomes of the interviews and local priorities for improvement. Stakeholders included managers, health and social care professionals, local policy officers and, in some cases, representatives of older people and informal carers, purchasers, regulators and regional health authorities. The number of workshop meetings depended on local needs and preferences. Research partners attended all workshop meetings and took minutes. Furthermore, a collaboratively designed protocol was developed to guide these workshops in order to ensure a uniform approach across all sites participating in SUSTAIN.

Information collected throughout the preparation phase was stored in a secure online database. Uniform templates were used to analyse and report the baseline information for each integrated care site [2].

Development phase: Based on the local improvement priorities identified in the preparation phase, steering groups consisting of local stakeholders were set up in each participating site. Starting in spring 2016, these steering groups designed improvement activities that were tailored to local priorities and addressed SUSTAIN's core domains. Information on these improvement activities was collected in two ways:

1. Improvement and implementation plans: A template for a structured and uniform description of the improvement and implementation plans was developed and used across the sites. This template included information on the sites' improvement objectives, improvement activities, expected outcomes, stakeholders involved and necessary resources. Templates were completed by the research partners collaborating with the sites after the steering group had agreed on the improvement project.
2. Flowcharts: For each site, flowcharts were developed to depict the pre-existing and improved pathways. Flowcharts were developed by research partners collaborating with the sites and included existing, new and modified actions, stakeholders, materials and processes needed to realise the proposed improvements.

Implementation phase: Starting in autumn 2016, participating integrated care sites implemented their improvement activities during the 18-month implementation phase. Table 1 shows the set of quantitative and qualitative data collection tools that was developed in order to monitor and evaluate implementation progress and impact on SUSTAIN's core domains. Data were collected at agreed and specified times and participants included health and social care professionals, managers from the integrated care service, older people using the integrated care service and (informal) caregivers of these older people. Data collection tools developed by SUSTAIN research partners were provided in English and subsequently translated into the national languages. Regular meetings and teleconferences took place between partners in order to standardise methods of data collection in each country.

Collected data were stored in a secure online database, for which strict data entry guidelines were developed and shared across research partners. To build the individual case studies, data were analysed using a three-staged approach, described in De Bruin et al., 2018 [3]:

1. Step 1: Each individual data source was analysed using either quantitative or qualitative methods, as appropriate for that specific data source. In order to standardise data analysis across all sites, uniform data analysis templates were developed based on discussions among research partners.

2. Step 2: After analysing each individual data source, results for that source were reduced to a series of summaries (in case of quantitative data) and thematic statements (in case of qualitative data). These summaries and thematic statements were provided in English.
3. Step 3: For each case study, summaries and thematic statements were amalgamated and underwent a process of pattern-matching across the data. In order to guide this process and ensure uniformity in data analyses across all case studies, an analysis framework was developed and used by all research partners. Data were analysed against two propositions and five analytical questions:
    - Proposition 1: Integrated care activities will maintain or enhance person-centeredness, prevention-orientation, safety, efficiency and coordination in care delivery.
    - Proposition 2: Explanations for succeeding in improving existing integrated care sites will be identified.
    - Analytical question 1: What seems to work, in what kind of situation, and with what outcomes when making improvements to integrated care?
    - Analytical question 2: What are the explanations for succeeding and improving integrated care sites?
    - Analytical question 3: What are the explanations for NOT succeeding and improving integrated care sites?
    - Analytical question 4: Are there any factors that are particularly strong in your analysis that could be seen as having an impact on integrated care improvements?
    - Analytical question 5: What factors can you identify in your site analysis that could apply to integrated care improvements across the EU, and be transferable?

Results of these analyses were reported in individual case study reports [4-10].

Table 1. Qualitative and quantitative measures to monitor and evaluate improvement progress and outcomes, adapted from de Bruin et al. (2018) [1]

**Data collection tool**	**Short description**	**Collection moment**
**SURVEYS**		
Socio-demographics of older people (users)	Survey developed by SUSTAIN researchers including information on age, gender, education, marital status, living situation and medical conditions	Recruitment and collection took place throughout implementation period
Socio-demographics of informal carers	Survey developed by SUSTAIN researchers including information on age, gender, education, marital status, relationship and distance to user, paid work and caregiving activities	Recruitment and collection took place throughout implementation period
Socio-demographics of professionals	Survey developed by SUSTAIN researchers including information on age, gender, nationality and occupation	Collection took place at the beginning and end of implementation period
Socio-demographics of managers	Survey developed by SUSTAIN researchers including information on age, gender,	Collection took place at the beginning and end of

Data collection tool	Short description	Collection moment
	nationality and occupation	implementation period
The Person Centred Coordinated Care Experience Questionnaire (P3CEQ)[11]	Survey measuring older people's experience and understanding of the care and support they have received from health and social care services	Recruitment and collection took place throughout implementation period
Perceived Control in Health Care (PCHC)[12]	Survey addressing older people's perceived own abilities to organise professional care and to take care of themselves in their own homes, and perceived support from the social network	Recruitment and collection took place throughout implementation period
Team Climate Inventory – short version (TCI-14) [13]	Survey measuring vision, participative safety, task orientation and experienced support for innovation of the improvement team	Collection took place at the beginning and end of implementation period
**INTERVIEWS**		
Semi-structured interviews with older people and/or their informal caregivers	Interview schedule developed by SUSTAIN researchers with items regarding users' and carers' perceptions of and experiences with the integrated care services and the extent to which they work in a person-centred, prevention-oriented, safe and efficient manner	Recruitment and collection took place throughout implementation period
Group interview with participating health and social care professionals	Interview schedule developed by SUSTAIN researchers with items regarding professionals' perception of and experiences with the improvement process, its facilitating and impeding factors and the extent to which it impacted person-centeredness, prevention-orientation, safety and efficiency of their way of working	Collection took place at the end of implementation period
Semi-structured interviews with managers	Interview schedule developed by SUSTAIN researchers with items regarding managers' perception of and experiences with the improvement process, its facilitating and impeding factors and the extent to which it impacted person-centeredness, prevention-orientation, safety and efficiency of their way of working	Collection took place at the end of implementation period
**OTHER TOOLS**		
Analysis of older people's care plans (when sites did not work with care plans, information was retrieved from clinical notes or other documentation)	Template developed by SUSTAIN researchers for predetermined content analysis of care plans, extracting information regarding needs assessments, goal-setting, medication reviews, falls, hospital and emergency admissions and advice on medication, safety and self-management	Recruitment and collection took place throughout implementation period
Time sheets	Template developed by SUSTAIN	Collection halfway through

Data collection tool	Short description	Collection moment
	researchers to collect information from staff regarding the number of hours dedicated to the improvement activities and costs of additional equipment and technology	and at the end of implementation period
**PROCESS INFORMATION**		
Steering group minutes	Minutes cover processes, discussions, decisions and contextual issues impacting on outcomes and implementation progress	Collection took place throughout development and implementation periods
Field notes	Field notes cover the researchers' notes and reflections on implementation progress	Collection took place throughout development and implementation periods

References

1. de Bruin SR, Stoop A, Billings J, Leichsenring K, Ruppe G, Tram N, et al. The SUSTAIN Project: A European Study on Improving Integrated Care for Older People Living at Home. Int J Integr Care. 2018;18(1):6.
2. Arrue B, Ambugo EA, Baan C, Barbaglia G, Billings J, de Bruin S, et al. Integrated care for older people living at home: current situation and ambition of sites participating in the SUSTAIN project. Wosko P, Ruppe G, editors. Vienna: Austrian Interdisciplinary Platform on Ageing / OEPIA; 2016.
3. De Bruin SR, Stoop A, Baan CA, Nijpels G, Billings J. Sustainable tailored integrated care foro lder people in Europe (SUSTAIN-project): lessons learned from improving integrated care in Europe. Bilthoven, Amsterdam, Canterbury: National Institute for Public Health and the Environment (RIVM), VU University Medical Center (VUmc), University of Kent; 2018.
4. Hoffmann H, Kamann D, Drews J, Claußen J. Sustainable tailored integrated care for older people in Europe (SUSTAIN-project): lessons learned from improving integrated care in Germany. Hamburg: Stiftung Gesundheit Fördergemeinschaft e.V., KV RegioMed Zentrum Templin, Pflegewerk Berlin; 2018.
5. De Bruin SR, Lemmens L, Baan CA, Stoop A, Lette M, Boorsma M, et al. Sustainable tailored integrated care for older people in Europe (SUSTAIN-project): lessons learned from improving integrated care in the Netherlands. Bilthoven/Amsterdam/Utrecht: National Institute for Public Health and the Environment (RIVM), VU University Medical Center, Vilans; 2018.
6. Billings J, Gadsby E, MacInnes J. Sustainable tailored integrated care for older people in Europe (SUSTAIN-project): lessons learned from improving integrated care in the United Kingdom. Canterbury: University of Kent; 2018.
7. Rull M, T T, Vainre M, Paat Ahi G. Sustainable tailored integrated care for older people in Europe (SUSTAIN project): lessons learned from improving integrated care in Estonia. Tallinn: Praxis Centre for Policy Studies Foundation 2018.
8. Reynolds J, Masana L, Cayuelas Mateu N, Espallargues Carreras M. Sustainable tailored integrated care for older people in Europe (SUSTAIN-project): lessons learned from improving integrated care in Catalonia (Spain). Barcelona: Agency for Health Quality and Assessment of Catalonia (AQuAS); 2018.
9. Häusler C, Ruppe G. Sustainable tailored integrated care for older people in Europe (SUSTAIN-project): lessons learned from improving integrated care in Austria Vienna: Austrian Interdisciplinary Platform on Ageing / OEPIA; 2018.
10. Ambugo E, Hoel V, Hagen T. Sustainable tailored integrated care for older people in Europe (SUSTAIN-project): lessons learned from improving integrated care in Norway. Oslo: University of Oslo; 2018.
11. Sugavanam T, Fosh B, Close J, Phil, Byng R, Horrell J, et al. Co designing a Measure of Person-Centred Coordinated Care to Capture the Experience of the Patient: The Development of the P3CEQ2018.
12. Claassens L, Terwee CB, Deeg DJH, Broese van Groenou MI, Widdershoven GAM, Huisman M. Development and validation of a questionnaire assessing the perceived control in health care among older adults with care needs in the Netherlands. Quality of Life Research. 2016;25(4):859-70.
13. Kivimaki M., Elovainio M. A short version of the Team Climate Inventory: Development and psychometric properties. Journal of Occupational and Organizational Psychology. 1999;72(2):241-6.
